# A Promising Mortar Produced with Seawater and Sea Sand

**DOI:** 10.3390/ma15176123

**Published:** 2022-09-03

**Authors:** Zhigang Sheng, Yajun Wang, Dan Huang

**Affiliations:** 1 Key Laboratory of Building Collapse Mechanism and Disaster Prevention, Institute of Disaster Prevention, China Earthquake Administration, Sanhe 101601, China; 2 Zhejiang Key Laboratory of Offshore Marine Engineering Technology, Zhoushan 316002, China; 3 School of Marine Engineering Equipment, Zhejiang Ocean University, Zhoushan 316002, China; 4 College of Mechanics and Materials, Hohai University, Nanjing 210098, China

**Keywords:** environment-friendship, mortar, seawater and sea sand, strength and damage

## Abstract

The aim of the study is the deep understanding of the essential reactivity of the environmentally friendly mortar by which its applicability can be justified. Created in the study was the environmentally friendly mortar, which helped relieve the increasing requirements on conventional building materials that are produced from exhausted freshwater and river sand nowadays. Seawater (SW) and sea sand (SS) collected from the Eastern Seas of China were used to produce the mortar at various ages, including 10-day, 33-day, and 91-day. Both the curing and working conditions of the mortar were natural marine ones. The physicochemical-mechanical behaviors were investigated using uniaxial compression tests (UCTs), Energy Dispersive Spectrometer (EDS), X-ray Diffraction (XRD), and thermal-field emission scanning electron microscopy (SEM) analysis to understand the essential reactivity of the mortar with age accumulation. The results indicated that hydration products and favorable components were generated promisingly in the mortar: the C-S-H (*x*CaO·SiO_2_·*z*H_2_O) development was certainly achieved in the critical environment during the curing and working period; the extensive generation of C-A-S-H (CaO·Al_2_O_3_·2SiO_2_·4H_2_O) helped densify the C-S-H grid, which caused the promising development of the uniaxial compression strength (UCS); the framework porosity of the mortar was restrained effectively due to the development of Friedel’s salt that re-bonded the interfacial cracks between SS and the hydration products with the age accumulation in the critical environment. Consequently, UCS and the resistance against damage of the mortar showed increasing behavior even in the critical environment. The study established Friedel’s salt working models and strength and damage models to interpret the physicochemical reactivity of the mortar as: the source of the strength and toughness was the proper polymerization between the native saline components and the hydration product mixture generated throughout the production, curing, and application without the leaching phenomenon. The novel models and interpretation of the physicochemical reactivity ensured the applicability of the mortar produced with SW and SS in the critical environment.

## 1. Introduction

**Figure 1:**
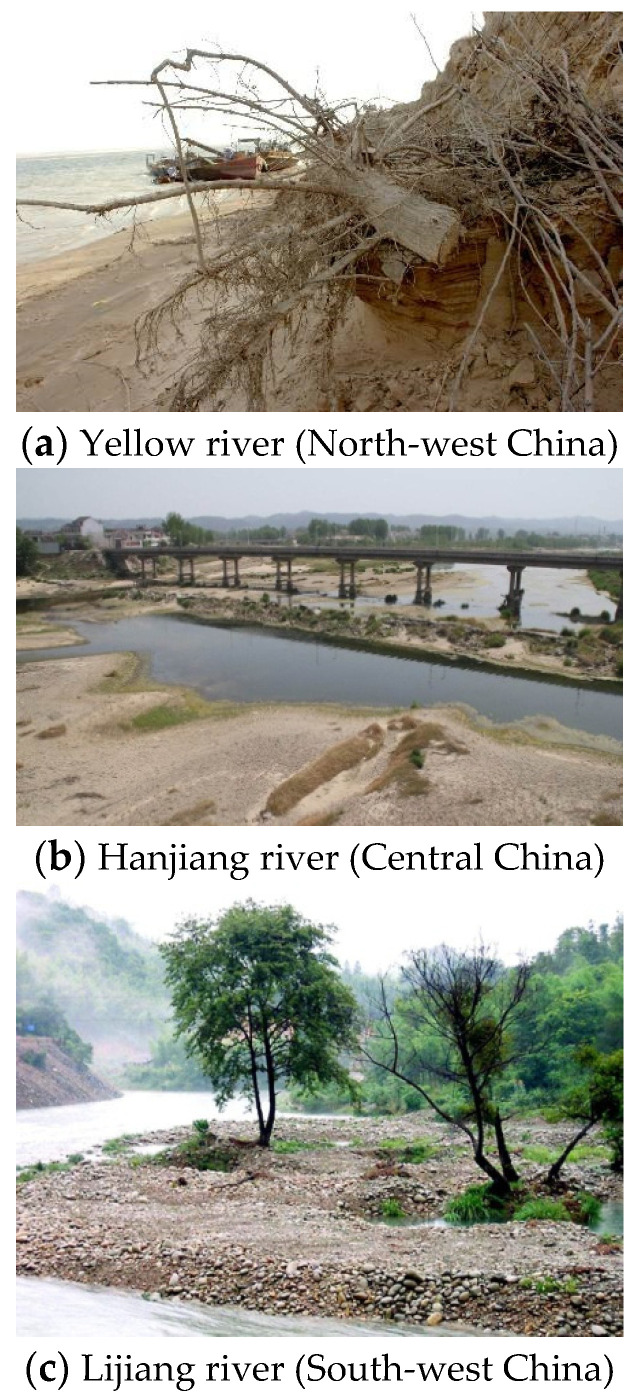
Wide-spreading disastrous cases due to river sand excavation in China.

Concrete and mortar are the most popular materials (i.e., cement-matrix materials) in infrastructure. However, the exhausting application of river sand has caused many ecological and environmental problems (Figure 1).

**Figure 2:**
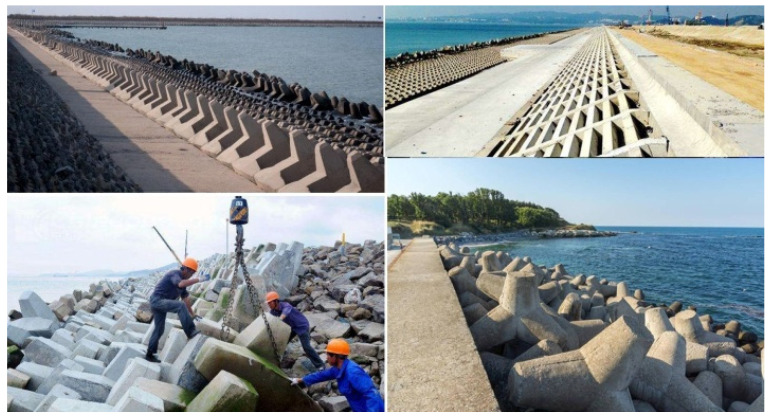
Sea embankment covers in the Eastern Seas of China; the blocks that composed the covers were produced with SW and SS without reinforcement.

Meanwhile, marine resource exploitation requires an efficient construction method that can reduce the freshwater application. The environment friendliness and sustainability were the main concepts that must be included in the novelty of the cement-matrix material composition. Hence, the applicability of seawater and sea sand has been the focus of construction and building materials, which have shown promising potential in many marine engineering cases. Particularly, without steel bars utilization, the direct application of seawater and sea sand in the production of cement-matrix materials achieved environmentally friendly and sustainable characteristics. Moreover, in China, the construction and building materials that used seawater and sea sand directly on-site showed increasing needs (Figure 2).

The work of Wang et al. [1] reported that the application of seawater and sea sand was a promising choice for marine structure construction. Their work also indicated that the cement-matrix materials using seawater and sea sand could offer adjustable performance by composition optimization. Karthikeyan and Nagarajan [2] studied the chloride quantification of sea sand and estimated the corresponding treatment in concrete. Dhondy et al. [3] produced concrete materials using seawater and sea sand under natural and unaltered curing conditions. They also investigated the short-term mechanical properties of the concrete using compressive tests. The results showed that the sea sand and seawater concrete held slightly higher strength at 28 days than conventional concrete. Suraksha et al. [4] presented two numerical approaches to study the durability of basalt fiber-reinforced polymer bars in seawater and sea sand concrete solutions subjected to various temperatures. The main goal of their work was to numerically predict the degradation of a basalt fiber-reinforced polymer bar in the marine environment. Sakthieswaran et al. [5] produced a polymer concrete modified using epoxy resin and sea sand. Their study results showed a significant improvement in the compressive and flexural strength due to the sea sand substitution in the polymer concrete. They also indicated that the application margin was unreinforced concrete. Bazli et al. [6,7,8,9,10] offered serial studies on fiber-reinforced polymer materials that were used in seawater and sea sand concrete; they also introduced glass-fiber-reinforced polymer composites using seawater and sea sand. The durability of the composites using seawater and sea sand was the focus. The damage mechanism of the fiber-reinforced polymer composite was also studied in their work. The serial work of Bazli et al. indicated that the mechanical performance of the concrete produced with seawater and sea sand showed no significant degradation under natural conditions. The proper treatment for the fiber-reinforced polymer materials even strengthened the concrete block. Ahmed et al. [11] also paid attention to the fiber-reinforced polymer material application in seawater and sea sand concrete. They pointed out that the appropriate mix design still achieved the needed strength and workability for the field concrete. Vafaei et al. [12,13] studied the sorptivity and microstructural behaviors of the high-strength fiber-reinforced seawater and sea sand concrete. Their work reported that the total porosity of the concrete showed finer characteristics. El-Khoury et al. [14] studied the effect of salinity, cement type, and specimen geometry on the degradation phenomenon of cement-based materials. Their work indicated that the salinity increase did not result in significant volumetric deformation in the specimens. El-Khoury et al. also reported the differentiation between conventional thoughts and their findings on the saline impact. According to the study, they confirmed that the sulfate attack from seawater environments was not the predominant one, and the pore-blocking effect of brucite and calcium carbonate formation near the surface slowed down the ionic diffusion into the cement matrix. Their study also indicated that the mechanical properties of the seawater-attacked specimens still remained equal to or slightly lower than the properties of the reference specimens. Seoung et al. [15] investigated the effects of seawater exposure on the mechanical, durability, and microstructural properties of Portland cement mortars. Their test results revealed that seawater exposure yielded positive effects, including flexural strength and durability improvements. Their interpretation was that the seawater curing, which filled specific size pores of 50–200 µm, generated the additional hydration, i.e., matrix densification. Weerdt et al. [16] studied the chloride ingress in Portland cement mortars exposed to seawater by comparing them with the ingress in mortars exposed to a NaCl solution. After 180 days of exposure to seawater, only the outer 1mm was enriched in sulfur and magnesium, which had only a limited impact on the chloride ingress. Particularly, the decalcification of C-S-H at the exposed surface was less severe. Palin et al. [17] tried to quantify the crack-healing capacity of seawater-submerged mortar, which was produced with blast furnace slag cement. Their study indicated that the cracks measuring 0.2 mm were fully healed after 28 days of submersion in seawater and the precipitation of the saline minerals from the carbonation in the cracks was principally responsible for healing. The study of Santhanam et al. [18] was focused on understanding the physical, chemical, and microstructural differences in sulfate attack from seawater and groundwater. They immersed the Portland cement mortars completely in solutions of seawater and groundwater. The results from their study reported that Portland cement mortars performed better in seawater in comparison with groundwater. Their study also indicated that the high Cl concentration of seawater played an important role by binding the C_3_A to form chloroaluminate compounds, including Friedel’s salt. Qu et al. [19] investigated the effects of seawater and undesalted sea sand on the hydration products, mechanical properties, and microstructures of Portland cement mortars. Their study showed that the saline content in sea sand accelerated the hydration reaction and increased the early-stage compressive strength. The addition of seawater could significantly accelerate hydration. The positive effect compensated for the side effect of sea sand and produced the cement mortar with seawater and sea sand with the highest compressive strength. Moreover, their study confirmed that the seawater application also upgraded the chemical property of C-S-H; chloride ions in both seawater and sea sand reacted with the C_3_A and CH to form Friedel’s/Kuzel’s salts, which accelerated the hydration reaction and optimized the microstructure of the mortars. The merit of the study of He et al. [20] was the justification of the positive effect of the carbonation in the seawater environment. The mortar using ordinary Portland cement and ion chelator was produced in their study. The investigation of the self-healing behavior of the mortar immersed in seawater showed that calcium carbonate was the key crack-healing product. Zhang et al. [21] evaluated the performance of alkali-activated mortars using seawater and sea sand. Their study indicated that the existence of seawater and sea sand promoted the formation of the C-S-H gel phase. Their mortar also showed higher compressive and flexural strengths at an early age. Zhang et al. interpreted that the natural internal curing effect and the dense interfacial transition zone in the mortar were the main causes. The study of Li et al. [22] confirmed that the alumina-rich cementitious materials effectively improved the chloride binding capacity of mortar mixed with seawater and sea sand. The study by Qin et al. [23] also verified the positive effect of the carbonation in the seawater environment. The results from their study reported that the products from the carbonation in the seawater cement mortar played a key role in hydration acceleration through the chemical and crystal nuclei mechanisms. There still exists, however, the crucial subject of how the essential reactivity of the marine-cementitious materials develops in the critical environment. The subject is determined to establish the marine epoch of material science and technology. The experimental study in this paper was conducted to explore the essential reactivity of the mortar produced with SW and SS. The physicochemical-mechanical experimental art was established in the study to interpret the mortar performance. UCTs were carried out in the study to explore the macro-strength and macro-damage characteristics of the mortar at various ages, which reported the mechanical properties. Moreover, the physicochemical behaviors of the mortar were investigated by EDS, XRD, and SEM, which helped understand the physicochemical mechanism. The relationship between the mechanical properties and physicochemical mechanism was discussed in the paper. The key novelty of the study is just the deep understanding of the essential reactivity derived from the physicochemical-mechanical behaviors, which ensures the rational application of the cement-matrix materials using SW and SS, including the mortar [24].

## 2. Materials and Methods

### 2.1. Mortar Composition

The study ideology and design composition of the mortar (Table 1) were conceptualized at the School of Marine Engineering Equipment of Zhejiang Ocean University (in Zhoushan, China). The mortar specimens in the study were produced in the Zhejiang Key Laboratory of Offshore Marine Engineering Technology of Zhejiang Ocean University (in Zhoushan, China).The key gravity ratios included SW:cement = 0.458:1 and SS:cement = 0.757:1.

The following information was offered to report the characteristics of the parental materials in detail.

*SW:* The mortar specimens were prepared with SW that was collected from the Eastern Seas of China. The key ions and the saline minerals in SW included: Cl^−^ at 17,552.126 ppm (i.e., mg/kg), Na^+^ at 8704.95 ppm, Mg^2+^ at 962.494 ppm, K^+^ at 466.411 ppm, Ca^2+^ at 314.85ppm, SO_4_^2−^ at 2650.5534 ppm, MgCl_2_ at 3774.72 ppm, CaSO_4_ at 1070.49 ppm, K_2_SO_4_ at 1040.46 ppm, and Br^−^ at 32.7351 ppm.

**Figure 3:**
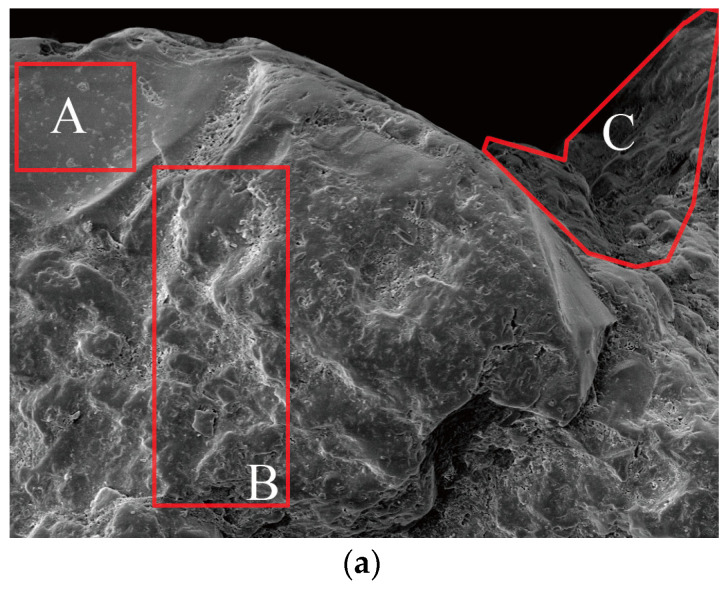

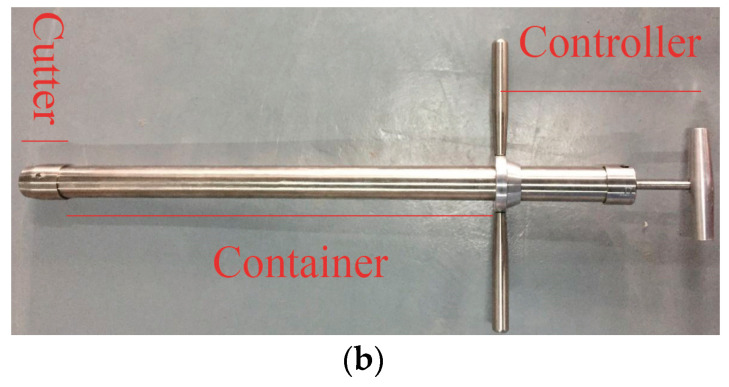
SS and driller. (**a**) SS image (Zone A was the SiO_2_ matrix; Zone B was the Cl^−^ complex; Zone C was the saline minerals of K^+^, Al^3+^, and Na^+^). (**b**) Driller (Cutter helped break the consolidated cover on the shelf; container carried the SS particles; controller was operated by human or machine to press the driller into the deep SS layer).

**Figure 4:**
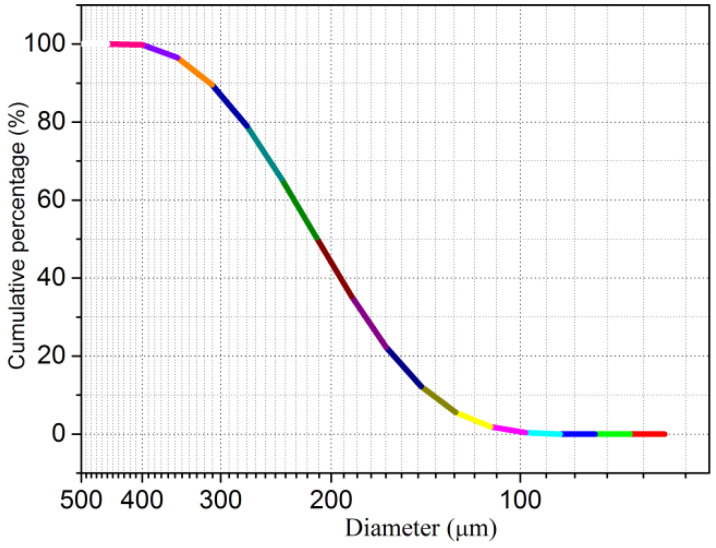
Size distribution of SS.

*SS:* SS was drilled from Dongsha shore of the Eastern Seas of China (Figure 3). An SEM image indicated that SS from Dongsha shore achieved superior roughness, which ensured the reliable interfacial cohesion between the SS particle’s faces and the components and hydration products mixture of the mortar. Furthermore, the SS used in the study was composed of SiO_2_ matrix, Cl^−^ complex, and the saline minerals of K^+^, Al^3+^, and Na^+^. Due to the dense distribution of the Cl^−^ complex and saline minerals on the particle’s faces, anion-cation exchange would happen between the paste and SS, which also contributed to the promising physicochemical reactivity of the mortar, even in the critical environment, i.e., the marine environment. The size distribution of SS was explored by Malvern Mastersizer 2000 analyzer (Malvern Panalytical Ltd., Malvern, UK) (size range of 2 × 10^−5^ mm–2 mm), and the results on the cumulative percentages are offered in Figure 4.

Based on size distribution, it was proposed that SS from Dongsha shore had uniform and discontinuous characteristics. Therefore, it was deduced that the discontinuousness of SS from Dongsha shore could offer abundant fill space for the components and hydration products mixture.

**Figure 5:**
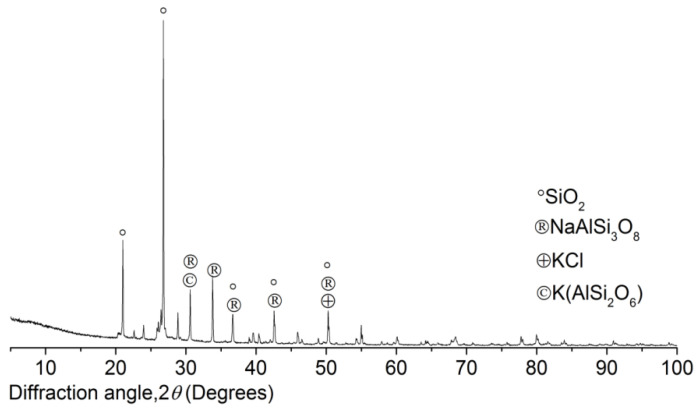
XRD patterns of sea sand.

The study also invited the XRD analysis (BRUKER AXS Inc., Madison, WI, USA) to investigate the SS mineral components, and the results are reported in Figure 5.

XRD patterns of the SS from Dongsha shore certainly showed the marine characteristics, and the saline minerals on the particles’ faces determined the specific performance of the mortar. The SiO_2_ matrix was covered densely by the saline minerals and Cl^−^ complex. According to the results from XRD, KCl was the predominant Cl^−^ complex; the main saline minerals included NaAlSi_3_O_8_ and K(AlSi_2_O_6_). Moreover, the anion-cation exchange between the paste and SS contributes to the physicochemical reactivity of the mortar during the curing and working period in the marine environment.

**Figure 6:**
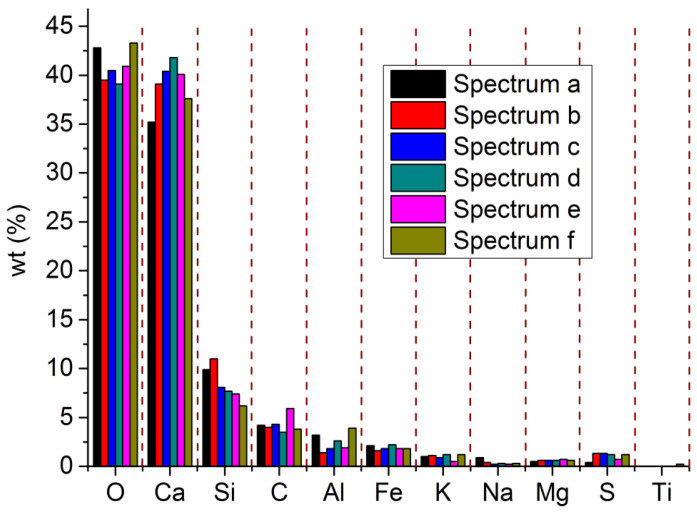
EDS spectrums of composite Portland cement P.C 42.5 R.

**Figure 7:**
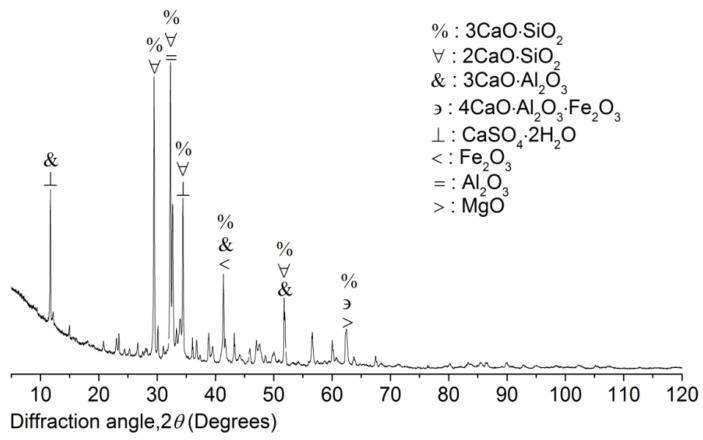
XRD patterns of composite Portland cement P.C 42.5 R.

*Cement* The composite Portland cement P.C 42.5 R (Hailuo Cement Co. Ltd., Zhangjiagang City, China) was used in the study to produce the mortar of SW and SS. The specification standards of the composite Portland P.C 42.5 R from CNSMC (Beijing, China) and ASTM were adopted because the two standards had consistency in the physicochemical quality of the composite Portland P.C 42.5 R and inspired the main concept of the study. The chemical composition of the composite Portland P.C 42.5 R was studied by EDS and XRD. EDS investigated the element content (EC) with five spectrums (Figure 6). The spectrums showed obvious consistency in terms of EC. According to the EDS results on the composite Portland cement P.C 42.5 R used in the study, the EC of Al was higher than that of Fe. Hence, it was deduced that the cement was rich in Al^3+^ complexes, including 3CaO·Al_2_O_3_ and 2CaO·Al_2_O_3_and poor in Fe^2+^ and Fe^3+^ complexes, including 4CaO·Al_2_O_3_·Fe_2_O_3_ and FeO. Based on XRD results (Figure 7), the main components of the composite Portland cement P.C 42.5 R used in the study included CaO, SiO_2_, Al_2_O_3_, FeO, 2CaO·SiO_2_, 3CaO·SiO_2_, 3CaO·Al_2_O_3_, and 4CaO·Al_2_O_3_·Fe_2_O_3_. Therefore, the results of XRD also justified the deduction from EDS. Meanwhile, 3CaO·Al_2_O_3_ existed at six dominant peaks in XRD patterns. By contrast, 2CaO·Al_2_O_3_ existed at four dominant peaks. Therefore, the predominant chemical component of the composite Portland cement P.C 42.5 is 3CaO·Al_2_O_3_, which will accelerate the early 180-day hydration. Hence, it is deduced that the mortar produced with the composite Portland cement P.C 42.5 in the study will reach its compressive strength peak at an early age younger than 180 days. FeO and 4CaO·Al_2_O_3_·Fe_2_O_3_ lived at one low peak in XRD patterns. Fe^2+^ and Fe^3+^ complexes were not the predominant chemical components. Hence, the results from EDS and XRD showed the exact consistency.

### 2.2. Experiments’ Standards, Equipment, and Methods

The Sino standards were the predominant references, and the American codes were also invited partly during the composition design and the mortar preparation.

*Mixing* [25,26]. The mortar paste was prepared in the specific mixer, and the type was UJZ-15 JGJ-90 (maximal power 1.5 kW and capacity 28 L) produced by Xinke company in Shijiazhuang, China. The standard preparation time was 10 min.

*Molding* [27]. The mortar paste was homogeneously filled into the normal molds (length × width × height = 70.7 × 70.7 × 70.7 mm) that were set on the vibro-machine for vibrating compaction. The key parameters included a vibrating period of 20 min, a vibrating frequency of 45 ± 5 Hz, and a vibrating amplitude of 0.3 mm.

The cubic specimens in the normal molds were kept indoors for 24 h, and the temperature and percentage relative humidity (RH) were 24 ± 5 °C and 85 ± 5%. The cubic specimens were then unloaded from the normal molds when they achieved the initial strength.

*Curing* [26,27,28]. The cubic specimens achieving the initial strength were retrieved into the curing chamber where the specific temperature and percentage relative humidity (RH) were 20 ± 5 °C and 92 ± 5%. Furthermore, the cubic specimens were immersed into the tank full of SW that was collected from the Eastern Seas of China, which could imitate the marine environment. The curing ages for the cubic specimens in SW included 9, 32, and 90 days, and the total ages, including the indoor resting period, correspondingly, were 10, 33, and 91 days. The paper referred to the total ages for the following discussion.

*Uniaxial compression system* [29,30]. The poly-tetra-fluoroethylene (PTFE) plates that were painted with silicon-based grease were used to reduce the shearing friction between the cubic specimens’ faces and the apparatus, by which the uniaxial compression load was ensured. The uniaxial compression system parameters are reported in Table 1.

## 3. Results and Discussion

Based on the mortar composition designed in this paper, the study produced three groups of cubic specimens, the total of which was nine. The physicochemical-mechanical experimental art was established in the study to explore the mortar mechanism, and the key results were reported as follows.

### 3.1. Mathematics and Discussion on UCT

The study invited the nominal stress in Equation (1) to quantify the UCS, which helped investigate the mechanical-physical performance of the mortar.

(1) $$S=\frac{f_{u}}{a_{0}}$$

In Equation (1) $f_{u}$ represents the maximal load of the uniaxial compression system and $a_{0}$ is the non-damaged cross-sectional area of the cubic specimen, the value of which is 70.7 × 70.7 mm^2^.

**Figure 8:**
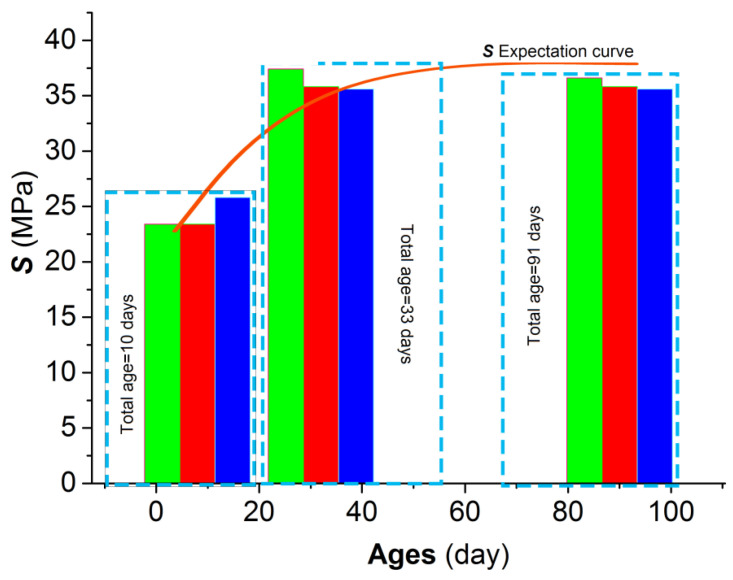
*S* distribution from UCTs.

Figure 8 demonstratesthe numerical distribution of *S* based on Equation (1) and the results from UCTs on nine cubic specimens.

According to the results from UCTs on three groups of the cubic specimens, the *S* expectation of the mortar with an age of 91 days was above 36 MPa. In terms of the expectation, the *S* of the cubic specimens with an age of 33 days reached the peak of 36.28 MPa; the *S* of the cubic specimens with an age of 91 days decreased by 0.7% in comparison with that of the 33-days-old; the *S* of the cubic specimens with an age of 91 days increased by 49% in comparison with that of the 10-days-old. Hence, the mortar compressive strength in the study generally showed increasing behavior during the working period in the marine environment. Moreover, the mortar’s compressive strength reached its peak at an early age of 91 days. Hence, the strength development of the mortar showed obvious consistency with the physicochemical quality of the composite Portland P.C 42.5 R, the predominant chemical component of which was 3CaO·Al_2_O_3_, which accelerated the early hydration and strength development. Furthermore, the strength development at an early age partly reduced the damage on the faces of the specimens caused by the salt petering, which impaired the toughness and resistance of the mortar faces. Therefore, the key conclusion of the study was that the mortar produced with SW and SS achieved different physicochemical-mechanical behaviors in comparison with the conventional building materials due to their different physicochemical reactivity.

**Figure 9:**
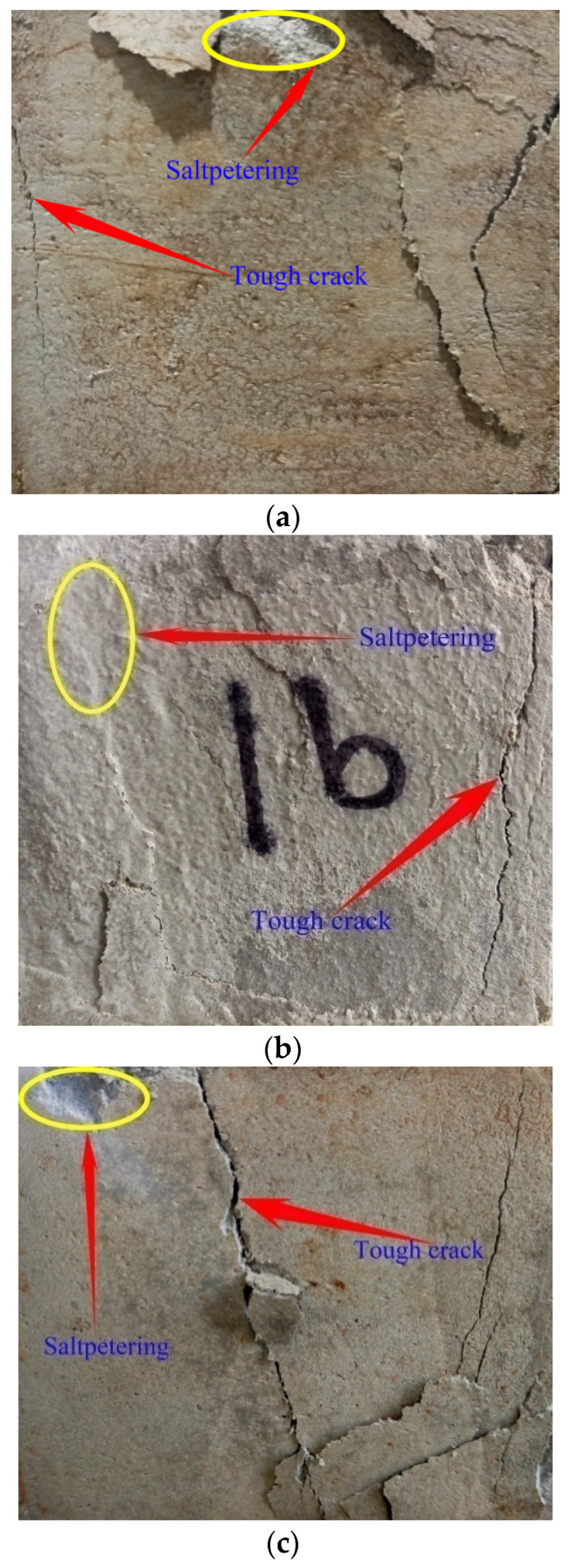
Damaged faces of cubic specimens. (**a**) 10-daysold(maximal width of tough cracks < 1 mm; there lived lower toughness development when the cubic specimen was damaged thoroughly). (**b**) 33-daysold (1 mm < maximal width of tough cracks < 2 mm; the mortar toughness developed certainly when the cubic specimen was damaged thoroughly). (**c**) 91-daysold (2 mm < maximal width of tough cracks < 3 mm; there lived the complete toughness development in the mortar when the cubic specimen was damaged thoroughly).

The salt petering (Figure 9a) generally existed in the mortar during the working period in the marine environment, which was the main cause of why the cubic specimens were damaged on the faces where the saline ions penetrated the framework of the mortar. Particularly, the microscopic young elastic cracks were activated due to the development of Friedel’s salt (3CaO·Al_2_O_3_·CaCl_2_·10H_2_O) in the young specimens.

**Figure 10:**
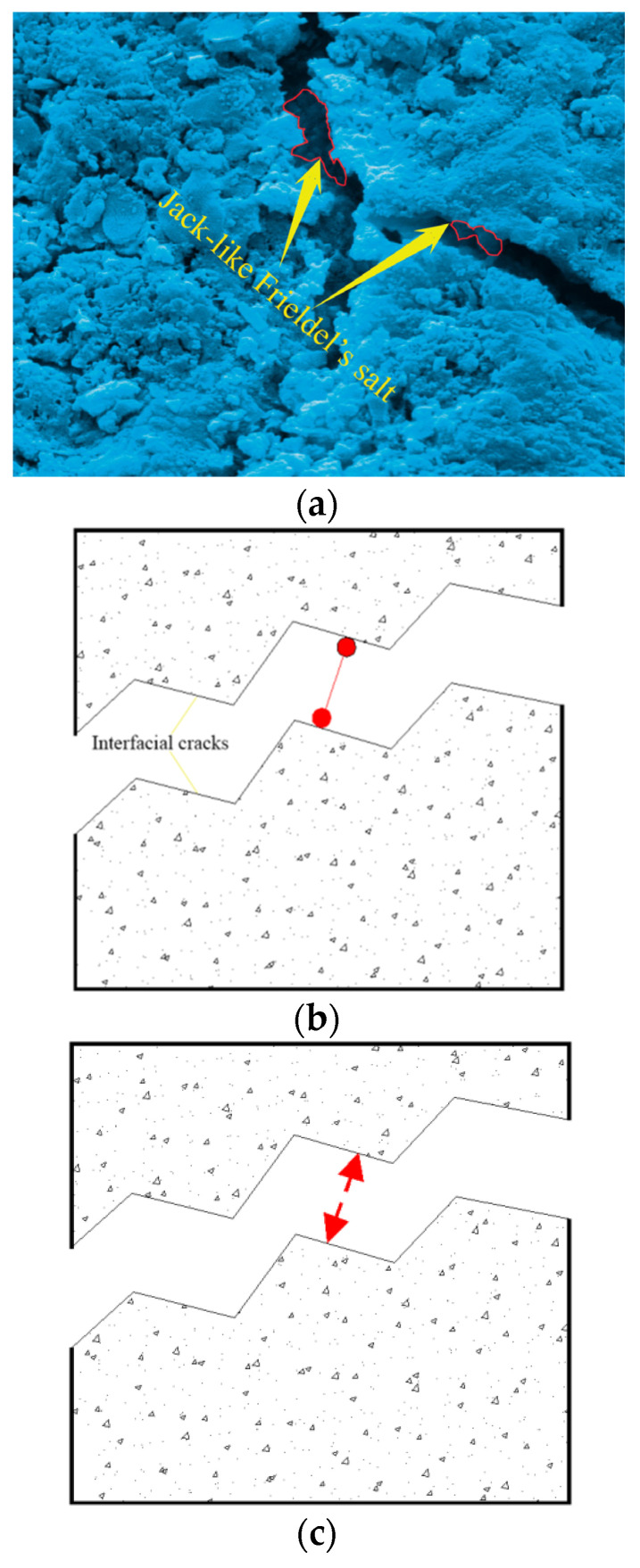
Friedel’s salt working model in young specimens.(**a**) SEM image of the 10-day-oldspecimen. (**b**) Jack working behavior of Friedel’s salt; the jack can be represented by a single-direction hinge. (**c**) Repulsive force model of Friedel’s salt with jack working behavior.

Friedel’s salt in young specimens played the jack role, lifting and breaking the interfacial cracks (Figure 10). Hence, the repulsive force was generated along the interfacial cracks, which activated the development of microscopic young elastic cracks and reduced the toughness and resistance of the mortar faces (Figure 10a); the depth of the young microscopic elastic cracks was below 3 μm, according to SEM results).

The jack-like Friedel’s salt and the interfacial cracks formed the statically determinate system. The interfacial cracks would evolve out of control once the statically determinate system was broken, which showed the linear-elastic feature. Consequently, the young specimens failed to generate wider tough cracks except for the microscopic young elastic cracks.

Moreover, in terms of the mortar, the 10-day UCS of which was above 23 MPa in the study, its physicochemical-mechanical behaviors also helped partly constrain the evolution of the microscopic young elastic cracks. The work of Xiao et al. also justified the early strength feature of the cementitious materials produced with SW and SS [31].

On the contrary, the development of Friedel’s salt was helpful for the grown specimens because most of the saline ions entered the internal grid of the grown specimens, the interfacial cracks of which were re-bonded due to Friedel’s salt’s polymerization effect in the marine environment. In the meantime, the open micro-pores in the framework were locked efficiently due to Friedel’s salt development in the grown specimens, which upgraded the mortar durability (Figure 9b,c).The specific physicochemical reactivity of the mortar produced in the study with SW and SS promisingly resisted the extreme case of seawater attack in terms of the cement type with >10% 3CaO·Al_2_O_3_ content. The key findings on Friedel’s salt polymerization effect were also justified partly by Vafaei et al. [12,13].

The glue-like Friedel’s salt and the interfacial cracks formed the statically indeterminate system. The interfacial cracks would evolve plastically with the gradual loss of the constraints from the statically indeterminate system, which showed the nonlinear-plastic feature. The grown specimens exactly generated the developed tough cracks during the failure process of the statically indeterminate system.

**Figure 11:**
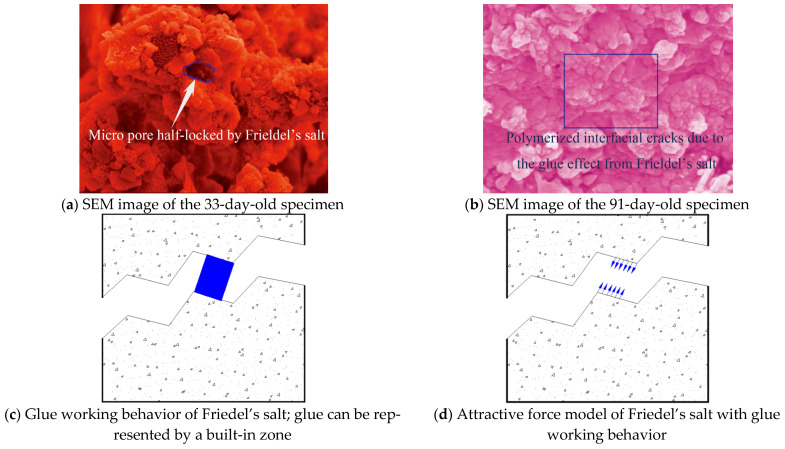
Friedel’s salt working model in grown specimens. Friedel’s salt in grown specimens played the glue role in polymerizing the interfacial cracks (**a**,**b**). Hence, the attractive force was generated along the interfacial cracks (**c**,**d**), which ensured higher toughness and resistance of the mortar under the uniaxial compression condition.

The typically damaged faces (Figure 9, Figure 10a and Figure 11a,b) from the cubic specimens indicated that the mortar toughness developed with age accumulation. The grown specimens generated wider tough cracks than the young ones under the uniaxial compression condition. The cubic specimens with wider tough cracks offered higher resistance against the load and damage development, which meant that the grown specimens developed superior performance to the young ones. Therefore, the mortar produced in the study achieved promising strength with age accumulation in the marine environment.

The physicochemical-mechanical behaviors of the mortar using SW and SS had close similarity with the pressure casting effect. Their key mechanism was the proper polymerization that could ensure the framework of the goal material was as high macroscopic integration and as low damage as possible. In terms of the mortar using SW and SS, the proper polymerization of Friedel’s salt in the interfacial cracks contributed promisingly to the toughness and resistance of the mortar without heavy damage to the framework. Meanwhile, the leaching phenomenon of the Ca^2+^ complex in the conventional mortar due to seawater attack did not happen in the mortar using SW and SS (Figure 11a,b). The framework of the hydration products mixture was polymerized properly by the native saline products, including Friedel’s salt.

**Figure 12:**
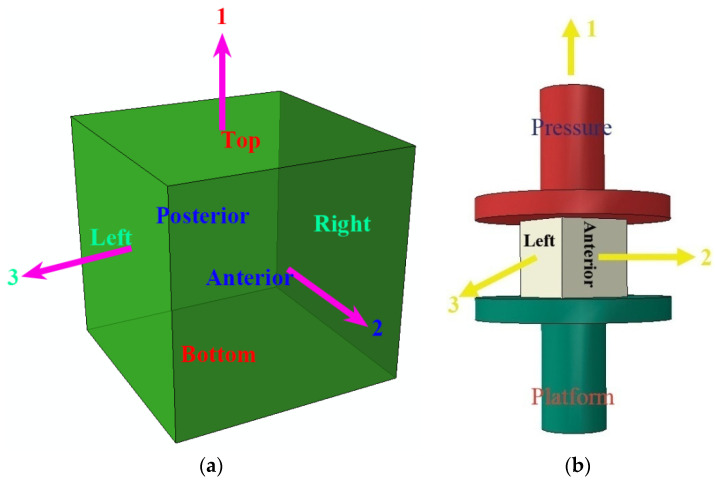
Strength and damage model in UCTs. (**a**) Coordinate system on the orthogonal damage tensor. (**b**) Cubic specimen position. 1 represented the first principal direction where the uniaxial compression load was applied and was formed by bottom and top faces of cubic specimen; 2 was the second principal direction and was formed by posterior and anterior faces; 3 designated the third principal direction and was formed by right and left faces.

Moreover, the orthogonal damage tensor Ω (Equations (2)–(5)) was created to interpret the mortar strength and toughness developed with age accumulation. Meanwhile, the cubic specimen position in UCTs was expressed in Figure 12.

(2) $$\Omega =\left[\begin{array}{ccc} \Omega_{1} & & \\ & \Omega_{2} & \\ & & \Omega_{3} \end{array}\right]=\left[\begin{array}{ccc} \frac{{w^{\prime }}_{1}}{w_{01}} & & \\ & \frac{{w^{\prime }}_{2}}{w_{02}} & \\ & & \frac{{w^{\prime }}_{3}}{w_{03}} \end{array}\right]$$

(3) $${w^{\prime }}_{1}=\max\left({w^{\prime }}_{-1},{w^{\prime }}_{+1}\right)$$

(4) $${w^{\prime }}_{2}=\max\left({w^{\prime }}_{-2},{w^{\prime }}_{+2}\right)$$

(5) $${w^{\prime }}_{3}=\max\left({w^{\prime }}_{-3},{w^{\prime }}_{+3}\right)$$

where 1, 2, and 3 indicated three principal directions (Figure 12a); uniaxial compression load was applied in direction 1, which was formed by bottom and top faces represented by −1 and +1, respectively; ${w^{\prime }}_{-1}$ and ${w^{\prime }}_{+1}$ designate the maximal widths of the tough cracks on bottom and top faces and ${w^{\prime }}_{1}$ is the result from the maximizing calculation on ${w^{\prime }}_{-1}$ and ${w^{\prime }}_{+1}$; directions 2 and 3 are the free wings of cubic specimen; direction 2 was formed by posterior and anterior faces represented by −2 and +2, respectively; ${w^{\prime }}_{-2}$ and ${w^{\prime }}_{+2}$ designate the maximal widths of the tough cracks on posterior and anterior faces and ${w^{\prime }}_{2}$ is the result from the maximizing calculation on ${w^{\prime }}_{-2}$ and ${w^{\prime }}_{+2}$; direction 3 was formed by on right and left faces represented by −3 and +3, respectively; ${w^{\prime }}_{-3}$ and ${w^{\prime }}_{+3}$ designate the maximal widths of the tough cracks on the right and left faces and ${w^{\prime }}_{3}$ is the result from the maximizing calculation on ${w^{\prime }}_{-3}$ and ${w^{\prime }}_{+3}$; $w_{01}$, $w_{02}$, and $w_{03}$ are the side lengths of cubic specimen under the initial state in directions 1, 2, and 3, respectively; the values of $w_{01}$, $w_{02}$, and $w_{03}$ in the study are 70.7 mm. All the tough cracks in the study were measured with a vernier caliper (0.1 mm).

The study reported the widths of the tough cracks of nine cubic specimens in Table 2**.** Meanwhile, the principal damage variables $\Omega_{i}$ (*i* = 1, 2, and 3) were computed based on Equation (2) and Figure 12b, and the results are offered in Table 2.

Consequently, the resistance tensor against damage ***R*** was defined as follows to interpret the macroscopic integration of the mortar developed with age accumulation [32].

(6) $$\begin{array}{cc} R & =\left[\begin{array}{ccc} R_{1} & & \\ & R_{2} & \\ & & R_{3} \end{array}\right]\\ & =\left[\begin{array}{ccc} \frac{S\varepsilon_{0}}{\left(1-\Omega_{1}\right)} & & \\ & \frac{S\varepsilon_{0}}{\left(1-\Omega_{2}\right)} & \\ & & \frac{S\varepsilon_{0}}{\left(1-\Omega_{3}\right)} \end{array}\right]\\ & =\left[\begin{array}{ccc} \frac{S\varepsilon_{0}}{\left(1-\left(\frac{{w^{\prime }}_{1}}{w_{01}}\right)\right)} & & \\ & \frac{S\varepsilon_{0}}{\left(1-\left(\frac{{w^{\prime }}_{2}}{w_{02}}\right)\right)} & \\ & & \frac{S\varepsilon_{0}}{\left(1-\left(\frac{{w^{\prime }}_{3}}{w_{03}}\right)\right)} \end{array}\right]\\ & =S\varepsilon_{0}\left[\begin{array}{ccc} \frac{1}{\left(1-\left(\frac{{w^{\prime }}_{1}}{w_{01}}\right)\right)} & & \\ & \frac{1}{\left(1-\left(\frac{{w^{\prime }}_{2}}{w_{02}}\right)\right)} & \\ & & \frac{1}{\left(1-\left(\frac{{w^{\prime }}_{3}}{w_{03}}\right)\right)} \end{array}\right] \end{array}$$

(7) $$\varepsilon_{0}=\frac{D}{w_{01}}$$

where *D* indicates the maximal displacement of cubic specimen in direction 1; $\varepsilon_{0}$ is the corresponding strain; *R_i_* (*I* = 1, 2, and 3) referred to the principal resistance variables against damage in directions 1, 2, and 3, respectively.

Based on the definition of ***R***, the nominal resistance scalar against damage *R*_n_ was calculated by Equation (8),

(8) $$R_{n}=\sum_{i=1}^{3}R_{i}$$

The study reported the values of the orthogonal damage tensor and resistance tensor against damage of nine cubic specimens in Table 2.

According to the results in Table 2, the grown specimens with wide, tough cracks achieved higher toughness than the young ones with narrow tough cracks. The results showed consistency with the work of El-Khoury et al. [14]. Furthermore, the grown specimens, when reaching their UCS *S*, had higher resistance against the load and damage than the young ones [33]. Coupled with the physicochemical reactivity, the grown specimens with higher ***R*** achieved superior macroscopic integration to the young ones. Therefore, the mortar performance under the uniaxial compression condition was interpreted as the evolution from the microscopic localization of Ω to the macroscopic integration of ***R***. Specifically, the promising macroscopic integration originated from the pressure casting effect due to which the framework of the mortar produced with SW and SS was densified by the proper polymerization of Friedel’s salt.

### 3.2. Results and Discussion on EDS

The study also invited EDS analysis (EDAX Inc., Mahwah, NJ, USA) to explore the mortar composition, and the results are reported in Table 3.

According to the EC of O and C, the oxidization and carbonization were developed during the working period of the mortar. Particularly, the carbonate complex would be polymerized in the study with the hydration products, which formed the framework of the mortar. The EC development of Al, Si, S, and Cl showed the summits at the age of 33 days. The EC of Mg, K, Na, Fe, and Ca decreased with age accumulation. The EC of Si showed the peak feature, which was produced at the age of 33 days. The development of Cl in the mortar produced in the study also created the EC summit at the age of 33 days.

### 3.3. Resultsand Discussion on XRD

**Figure 13:**
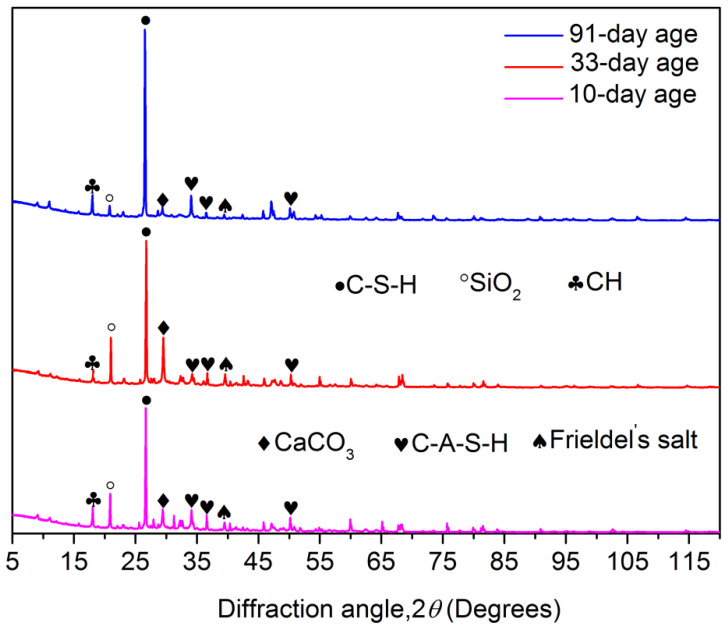
Mortar XRD patterns.

XRD patterns in the study demonstrated that C-S-H was the predominant hydration product (Figure 13). Particularly, the peak height of C-S-H ascended directly from 10-days old to 91-daysold. Therefore, C-S-H developed exactly with age accumulation, which indicated that the physicochemical reactivity of the mortar produced in the study was certainly growing during the working period in the marine environment. The development of CH (Ca(OH)_2_) showed the saddle feature: XRD reported the upgrowth of CH at 10-day-old and 91-day-olds; the peak height of CH at 33 days old showed a distinct drop. The C-A-S-H was another key hydration product in the mortar. Particularly, XRD patterns in the study exhibited the general existence of C-A-S-H. More peaks of C-A-S-H were identified in XRD patterns than that of CH. The generation of Friedel’s salt was also observed. According to XRD patterns, the growth of Friedel’s salt created its peak at 33 days old when the Cl EC summit was created consistently. The development of Friedel’s salt showed a reduction at 91 days old. The existence of the mixed salt (Na_2_SO_4_·H_2_O·5(CaSO_4_)·2H_2_O) in the mortar came from its low dissolvability. XRD patterns reported that SiO_2_ development reached the highest peak at 33 days old, which was consistent with the EDS results that indicated that the mortar generated the corresponding Si EC summit at this age. Furthermore, SS and the clinker were the predominant sources of SiO_2_. The mortar also generated the matrix of CaCO_3_.Correspondingly, the carbonate complex reinforced the framework of the mortar when polymerized with the other hydration products [34]. Based on the results from Xue, the formation of the carbonate complex restrained exactly in the saline environment the side effects of the micro-flaws in the framework of the cementitious materials.

### 3.4. Resultsand Discussion on SEM

**Figure 14:**
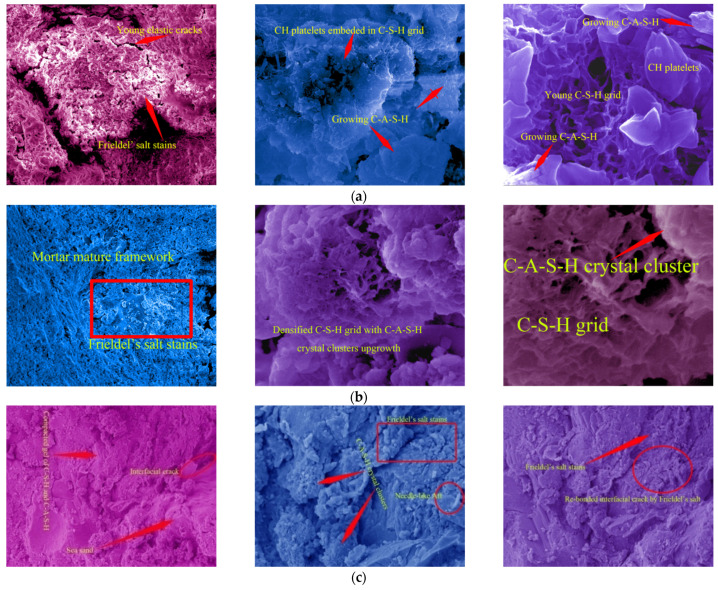
Mortar SEM images. (**a**) Results at 10 days old. (**b**) Results at 33 days old. (**c**) Results at 91 days old.

SEM images (Figure 14a, 10-days-old) showed the developing feature of the young mortar at 10 days old: Friedel’s salt was growing actively, and the mesoscopic salt petering was generated on the cubic specimens’ faces; C-A-S-H with lower density was developing around the C-S-H grid, the porosity of which displayed the loose structure at this age; the maximal size of micro-pores in the framework was 30 μm; the CH platelets were cultivated freely with lower compression from the framework. Therefore, the young mortar achieved only the tender framework, the hydration of which was not completed. With the free development in the micro-pores of the mortar, Friedel’s salt did not effectively offer the cohesive and locking function for the framework, except for the superficial salt petering. Correspondingly, the value of the mortar UCS at this age was lower than that at the grown age. However, the cement used in the study was rich in 3CaO·Al_2_O_3_. The development of C-A-S-H showed promising potential, which played a key role in the mortar strength increase with age accumulation.

With age accumulation, the gel of C-S-H and C-A-S-H created the mature mortar framework (Figure 14b, 33-day-old) in the marine environment where Friedel’s salt stains re-bonded the micro-flaws. The upgrowth of the C-A-S-H crystal clusters densified the C-S-H grid, which caused the promising development of the mortar strength (Figure 8). The maximal size of micro-pores in the framework was compressed to be 2 μm. Consequently, the micro-flaws in the framework were fused and locked by the developed Friedel’s salt, and the development of Friedel’s salt in the framework of the grown mortar hindered the dilation of the tough macroscopic cracks. Furthermore, the cation exchange, namely, the replacement of Si^4+^ and K^+^ on the SS particles faces by Al^3+^ and Ca^2+^ from the paste, raised the interfacial integrity of the mortar. In comparison with the 5μm microscopic young elastic cracks in the mortar at 10 days old, the interfacial cracks in the grown mortar were rehabilitated due to the cation exchange, and the width was below 1 μm. The marine characteristics of the SS used in the study were the main cause of the cation exchange. Therefore, the mortar strength in the study showed a generally increasing behavior during the working period in the marine environment. Meanwhile, the EC summits of Si, Al, and Cl were generated at 33 days old when the development of the hydration products and Friedel’s salt reached their peak. Hence, the compaction and cohesion from the hydration products and Friedel’s salt were achieved at an intense level. Correspondingly, the expectation of the mortar UCS attained the peak of 36.28 MPa at 33 days old; the ***R*** of the mortar at this age also achieved the highest level.

The SEM images of the mortar at 91 days old (Figure 14c) demonstrated that the gel of C-S-H and C-A-S-H was growing during the working period in the marine environment, and the gel layers were compacted significantly with the hydration development. Particularly, C-A-S-H strengthened the C-S-H grid, and the micro-pores in the mortar were filled with C-A-S-H crystal clusters. Although the interfacial cohesion between the SS particle’s faces and the gel of C-S-H and C-A-S-H was reduced partially due to the existence of Aft, the interfacial cracks were compressed with the upgrowth of the gel of C-S-H and C-A-S-H [35]. According to the work by Lu et al., the SW mixture certainly improved the microscopic structures of the cementitious materials. Meanwhile, due to the discontinuousness of the SS used in the study, the abundant fill space in the mortar framework could meet the free upgrowth of the gel of C-S-H and C-A-S-H without inducing destruction in the crystal clusters. Moreover, Friedel’s salt re-bonded the interfacial cracks between the SS and the hydration products in the marine environment with age accumulation. The porosity of the mortar was still densified at the grown age. The pressure casting effect from Friedel’s salt development offered the proper polymerization in the interfacial cracks without heavy damage to the framework. Therefore, the Cl^−^ seepage in the mortar was also restricted efficiently [36].

## 4. Conclusions

In this paper, UCTs were adopted to investigate the mechanical performance of the mortar produced with SW and SS. The physicochemical reactivity of the mortar was also explored with EDS, XRD, and SEM. The mathematical models were established to correspondingly interpret the physicochemical-mechanical experimental art. The following conclusions based on the work can be drawn:

1. The mortar produced in the study achieved the promising development of the components and the hydration products, including C-S-H, C-A-S-H, and CH, even under natural conditions. The physicochemical reactivity of the mortar showed increasing behavior in the marine environment due to the anion-cation exchange between the paste and SS, Friedel’s salt polymerization effect and the lasting development of hydration products with age accumulation. Furthermore, the physicochemical reactivity brought out the different physicochemical-mechanical behaviors of the mortar produced using SW and SS in comparison with conventional building materials.
2. Friedel’s salt generation caused the salt petering in the young mortar. However, the polymerization from Friedel’s salt helped re-bond the micro-flaws in the grown mortar. The cohesive and locking effect from Friedel’s salt contributed exactly to the interfacial integrity of the mortar framework. The leaching effect of the Ca^2+^ complex in the conventional mortar due to seawater attack was restrained absolutely by the help of the proper polymerization between the hydration products mixture and the native saline products in the mortar using SW and SS.
3. The interfacial integrity of the mortar produced in the study was also guaranteed by the generally existing cation exchange between the sea sand and the hydration products.
4. The C-A-S-H hydration product helped compact the mortar framework. The mortar was produced with cement rich in 3CaO·Al_2_O_3_, which ensured the promising development of the components and hydration products. The development of the components and hydration products reinforced the microscopic structure of the mortar. The early strength feature of the mortar helped constrain the salt petering and reduce the superficial damage. The depth of the microscopic young elastic cracks in the mortar was controlled below 3 μm, which ensured that the strength of the mortar produced in the study increased correspondingly in the marine environment.
5. The findings of the saline ions discussed in the current literature were re-estimated. Not including the addition of the bars, the chemical structure of the cement-matrix materials produced with SW and SS was solidified in the natural marine environment by saline polymerization, the development of which was ensured by the adsorption and adjustment in the hydration products.
6. Existing in the mortar using SW and SS throughout the production, curing and application was the proper polymerization between the hydration product mixture and the native saline products. Being similar to the pressure casting effect, the key mechanism of the proper polymerization was the time, magnitude, and state of the internal force generated by the native saline products in the interfacial cracks.
7. The grown mortar showed promising strength that developed in the marine environment with age accumulation. Coupled with the physicochemical reactivity, the grown mortar, with the increasing macroscopic integration that originated from the pressure casting effect, achieved higher resistance against the load and damage than the young ones. Particularly, the direct application of the mortar under natural conditions was the applicable choice.
8. The study of the direct application of the environmentally friendly mortar was another key job, the applicability of which was justified in this paper. The direct application of the environmentally friendly mortar created in the study reduced the requirement for the river sand, which will help control carbon emissions during the resource-devouring preparation of the river sand.

## Figures and Tables

**Table 1:** Uniaxial compression system parameters.

Extreme Load (kN)	Minimal Load Rate (kN/s)	Maximal Load Rate (kN/s)	Maximal Stroke of Vertical Main-Shaft (mm)	Return Stroke Velocity (mm/s)	Platform Area (mm^2^)	Power (kW)
300	0.5	30	260	15	1.47 × 10^4^	0.75

**Table 2:** Mortar damage and resistance characteristics.

Specimens	Direction 1			Direction 2			Direction 3			*Ω*_1_	*Ω*_2_	*Ω*_3_	*D*_1_	*ε*_max1_	*R*_1_	*R*_2_	*R*_3_	*R*_n_
	*w*′_−__1_	*w*′_+1_	*w*′_1_	*w*′_−__2_	*w*′_+2_	*w*′_2_	*w*′_−__3_	*w*′_+3_	*w*′_3_									
1	0.2	0.6	0.6	0.6	0.8	0.8	0.7	0.6	0.7	0.008	0.011	0.010	3.3	0.047	1.102	1.105	1.104	3.311
2	0.3	0.6	0.6	0.6	0.9	0.9	0.8	0.6	0.8	0.008	0.013	0.011	3.2	0.045	1.069	1.073	1.072	3.214
3	0.2	0.5	0.5	0.3	0.6	0.6	0.6	0.4	0.6	0.007	0.008	0.008	3.2	0.045	1.177	1.178	1.178	3.533
4	1.0	1.1	1.1	1.2	1.4	1.4	1.2	1.0	1.2	0.016	0.020	0.017	3.0	0.042	1.613	1.619	1.615	4.847
5	1.2	1.3	1.3	1.6	1.8	1.8	1.7	1.4	1.7	0.018	0.025	0.024	3.0	0.042	1.548	1.559	1.557	4.664
6	1.0	1.2	1.2	1.4	1.6	1.6	1.6	1.2	1.6	0.017	0.023	0.023	3.1	0.044	1.589	1.598	1.598	4.783
7	2.0	2.3	2.3	2.1	2.5	2.5	2.5	2.3	2.5	0.033	0.035	0.035	2.8	0.040	1.499	1.503	1.503	4.505
8	2.0	2.6	2.6	2.5	2.9	2.9	2.7	2.2	2.7	0.037	0.041	0.038	2.7	0.038	1.420	1.426	1.422	4.268
9	2.5	2.7	2.7	2.6	3.0	3.0	2.7	2.3	2.7	0.038	0.042	0.038	2.9	0.041	1.519	1.525	1.517	4.563
Unit	mm									/			mm	/	MPa			

**Table 3:** Elements’ content and distribution feature from EDS analysis.

Ages	C		O		Na	
(Days)	wt (%)	Feature	wt (%)	Feature	wt (%)	Feature
10	9.1	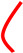	46.6	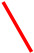	1.7	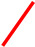
33	8.6		49.3		0.6	
91	8.68		55.7		0.56	
Ages	Mg		Al		Si	
(Days)	wt (%)	Feature	wt (%)	Feature	wt (%)	Feature
10	0.9	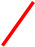	1.8	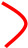	6.8	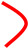
33	0.7		2.2		7.3	
91	0.5		0.98		4.47	
Ages	S		Cl		K	
(Days)	wt (%)	Feature	wt (%)	Feature	wt (%)	Feature
10	0.3	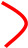	0.4	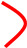	1.5	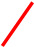
33	0.8		1		0.4	
91	0.56		0.23		0.24	
Ages	Ca		Fe			
(Days)	wt (%)	Feature	wt (%)	Feature		
10	29.1	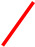	1.8	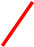		
33	28.2		1			
91	27.69		0.39			

## Data Availability

Not applicable.
